# PySWMM: The Python Interface to Stormwater Management Model (SWMM)

**DOI:** 10.21105/joss.02292

**Published:** 2020-08-04

**Authors:** Bryant E. McDonnell, Katherine Ratliff, Michael E. Tryby, Jennifer Jia Xin Wu, Abhiram Mullapudi

**Affiliations:** 1 EmNet, a Xylem Brand; 2 U.S. Environmental Protection Agency, Office of Research and Development; 3 Department of Civil and Environmental Engineering, University of Michigan

## Abstract

Stormwater management seeks to reduce runoff from rain or melted snow and improve water quality. Where it can absorb into soil, runoff is filtered and returns to streams, rivers, and aquifers, but in developed areas, precipitation often cannot soak into the ground because impervious surfaces (e.g., pavement, buildings), and already saturated soils can create excess runoff. This water, which can contain pollutants, then runs across urban surfaces and into storm drains, drainage ditches, and sewer systems. Stormwater runoff can cause flooding, erosion, infrastructure and habitat damage, and contamination (including combined and sanitary sewer overflows). In urban and developed areas, effective stormwater management that routes and detains stormwater helps to mitigate these impacts and improve water quality.

The U.S. Environmental Protection Agency’s (EPA) Stormwater Management Model (SWMM) is a dynamic rainfall-runoff model that has been used for decades to conduct single event or long-term hydrologic, hydraulic, and water quality simulations. It has been used widely throughout the world for planning, analysis, and design of drainage systems (Rossman, 2015). SWMM models the hydrologic processes that generate runoff including rainfall, snowmelt, evaporation, infiltration, and groundwater dynamics, and it routes water through a hydraulic network that includes channels, pipes, storage units, and pumps. The EPA distribution of SWMM does not, however, allow the modeler to interact with the SWMM model during simulation time nor access all of the simulated values and results.

Over the last decade, several libraries have been developed to read, parse, and run SWMM models (*.inp). These tools have been developed in several programming languages including, but not limited to, Python, R, MATLAB, and Visual Basic. Beyond simple SWMM interfacing utilities, many libraries include a collection of specific features for different applications. Some authors have focused on optimization frameworks (Macro, Matott, Rabideau, Ghodsi, & Zhu, 2019; Martínez-Solano, Iglesias-Rey, Saldarriaga, & Vallejo, 2016; Pathirana, 2014) and model calibration (Leutnant, Döring, & Uhl, 2019), and others have moved toward water quality modeling within their frameworks (Banik, Di Cristo, & Leopardi, 2014). Even others have extended functionality to support modeling of real-time controls (Riaño-Briceño, Barreiro-Gomez, Ramirez-Jaime, Quijano, & Ocampo-Martinez, 2016). From the most general standpoint, most of these previous works have one major thing in common – a wrapper interface is used to generate a new *.inp (SWMM input) file – which duplicates the data model, adding redundancy. From a maintainability standpoint, the scientific and engineering communities significantly benefit from directly accessing the SWMM data model during a simulation without having to read and write new *.inp files.

In support of the OpenWaterAnalytics (OWA) open source initiative, the PySWMM project encompasses the development of a Python interfacing wrapper to SWMM with parallel on-going development of the Open Water Analytics SWMM5 application programming interface (API). PySWMM (along with the co-development of the SWMM5-API) is being developed to enable Pythonic to access the SWMM data model, which facilitates rapid prototyping, and it also enables users to interact with a model during simulation time. Since PySWMM incorporates enhancements to the SWMM code base, it retains backward compatibilty and ultimately serves to augment what SWMM can do. Current mid-simulation capabilties include the ability to change hydraulic network settings, load externally-generated inflows, and maniuplate low impact development (LID) parameters. Node, link, subcatchment, LID statistics, and results are also accessible both during and after simulation time. PySWMM provides a single framework which encompasses a collection of low-level interfacing functions to the SWMM data model (getters and setters), which facilitates editing of network and hydrologic parameters. This functionality allows researchers and engineers to streamline stormwater model optimization, controls, and result post-processing in order to more effectively address scientific and engineering questions related to water runoff quantity and quality.

PySWMM is actively being used to address a range of topics in industry, academia, and in government. In industry, PySWMM has been used for real-time controls and asset optimization. It has been used in academia for studying the effects of real-time controls and model predictive controls of drainage systems (Li, Burian, & Oroza, 2019; Sadler et al., 2019). In the EPA’s Office of Research and Development, PySWMM is being used to model the precipitation-driven transport of contaminants and pollutants in urban environments (Ratliff, McDonnell, Tryby, & Mikelonis, 2018). EPA SWMM has been used widely for research applications, with applications being described in hundreds of journal articles and conference proceedings (Niazi et al., 2017). It is also used for developing discharge permits, and in addition, it is accepted by the Federal Emergency Management Agency for floodway analysis for National Flood Insurance Program purposes. PySWMM facilitates ease of use and enhances the capabilities of EPA SWMM for these research, engineering, and regulatory applications.

## Open Water Analytics Group

OWA is a group of individuals who have come together to collaborate and grow model extensibilty and features for a number of EPA models, including SWMM and EPANET (used for modeling drinking water distribution systems). We are an open community for the exchange of information and ideas related to computing and analysis in the water and wastewater industries and related research aims, and members with varying affiliations work together to craft the current and future development of these mission-critical software tools. The group has close relationships with the EPA and adheres to common coding practices that are in line with the direction of EPA. More information can be found on wateranalytics.org.
